# Using a Syrian (Golden) Hamster Biological Model for the Evaluation of Recombinant Anthrax Vaccines

**DOI:** 10.3390/life11121388

**Published:** 2021-12-11

**Authors:** Tatiana Kravchenko, Galina Titareva, Irina Bakhteeva, Tatiana Kombarova, Alexander Borzilov, Raisa Mironova, Kseniya Khlopova, Vitalii Timofeev

**Affiliations:** State Research Center for Applied Microbiology and Biotechnology (SRCAMB), 142279 Obolensk, Russia; tbkrav@mail.ru (T.K.); titarevag@mail.ru (G.T.); bahtejeva@mail.ru (I.B.); kombarova.tatyana@yandex.ru (T.K.); borzilov@obolensk.org (A.B.); mironovaraisa53@mail.ru (R.M.); xlopova.12@yandex.ru (K.K.)

**Keywords:** anthrax, *Bacillus anthracis*, Syrian hamsters, biomodels, vaccines

## Abstract

In this paper, we demonstrate that a Syrian hamster biological model can be applied to the study of recombinant anthrax vaccines. We show that double vaccination with recombinant proteins, such as protective antigen (PA) and fusion protein LF1PA4, consisting of lethal factor I domain (LF) and PA domain IV, leads to the production of high titers of specific antibodies and to protection from infection with the toxicogenic encapsulated attenuated strain *B. anthracis* 71/12. In terms of antibody production and protection, Syrian hamsters were much more comparable to guinea pigs than mice. We believe that Syrian hamsters are still underestimated as a biological model for anthrax research, and, in some cases, they can be used as a replacement or at least as a complement to the traditionally used mouse model.

## 1. Introduction

Anthrax is a disease caused by the Gram-positive, spore-forming bacterium *Bacillus anthracis*. It is well established that *B. anthracis* exhibits its pathogenic properties due to the presence of two plasmids encoding two main factors of pathogenicity. Plasmid pXO2 carries the genes of a poly-γ-D-glutamic acid capsule synthesis (the *capBCAD* operon). This capsule protects *B. anthracis* from the host’s immune response. Plasmid pXO1 carries genes encoding a three-component anthrax toxin, including a lethal factor (LF, metalloprotease), edema factor (EF, adenylate cyclase), and a protective antigen (PA, a non-toxic protein that binds to the host cell and ensures the transfer of LF and EF into the host cell). 

Anthrax has some selectivity, and it affects different animals with different efficacy. The most sensitive to anthrax are ungulate herbivores, and it is among them that the vast majority of cases of anthrax are recorded [1,2,3]. At the same time, it is the ungulates that form the basis of agriculture; therefore, any epidemics among them lead to significant economic losses and the risk of transmission of infection to humans. Although humans are less susceptible to anthrax than ungulates, the disease can be severe and fatal for humans [1].

Therefore, the study of the anthrax pathogenesis mechanisms, the determination of the *B. anthracis* strains’ biological properties, and the development of methods for preventative vaccines and treatment of livestock and humans anthrax remain urgent. However, almost all laboratories studying anthrax are forced to use in their work not ungulates and or primates imitating humans, but more accessible biological models, such as small rodents and rabbits. The use of ungulates and non-human primates is limited not only by bioethical considerations but also by economic reasons: the cost of the animals and their housing in BSL3-BSL4 vivariums make the use of such animals unreasonably expensive for routine work. However, rodents differ from ungulates and primates in the characteristics of the infectious process. Although the epidemiology of anthrax in wild rodents is practically unknown, they are more likely to be resistant to infection in natural conditions. The main argument in favor of this statement is the absence of death in wild and synanthropic rodents during anthrax outbreaks in herbivores, although these rodents feed on the same food as diseased herbivores and even eat the corpses of animals killed by anthrax [4]. Additionally, there are not many published works on the experimental infection of wild rodents. For example, as reported by Walker et al., 1967 [5] it is mentioned that an injection of *B. anthracis* spores to black rats did not lead to the development of anthrax in them. A larger-scale experiment described in [6] gave somewhat ambiguous results: 8 out of 12 studied species were susceptible to infection, 2 species were resistant, and 2 more showed too wide a dispersion of results to draw a conclusion. As for rabbits, we were unable to find any mention of natural (or even experimental) anthrax in wild lagomorphs, which also indicates that these animals do not suffer from anthrax in nature. The features of anthrax in wild animals are very interesting, but from a practical point of view, the same features are more important in laboratory animals, such as mice, rats, guinea pigs, and rabbits. Each of these species (and sometimes individual inbred lines within the same species) differs in sensitivity to anthrax and in the features of the infectious process. Therefore, researchers have to select a biological model for each specific task and sometimes use several species of animals in one experiment. A comprehensive description of the use of different laboratory animals for anthrax research can be found, for example, in a very detailed review by Welkos et al. [7]. The mouse model is most often used in routine work. These advantages of mice reduce the cost of experiments and make it possible to increase the number of animals used, which means an increase in the statistical reliability of the results obtained. In addition, mice are susceptible to infection with single-plasmid *B. anthracis* strains. Such strains are non-virulent to humans, and experiments with them can be carried out not only in the BSL3–4 laboratories. On the one hand, this greatly facilitates the design of the experiment, but, on the other hand, the results obtained can hardly be extrapolated to the anthrax of the main hosts and require confirmation using another biological model infected with two-plasmid strains. Rats are relatively resistant to infection, but at the same time, are very sensitive to the anthrax toxin; therefore, rats are used primarily to assess the effectiveness of toxin-neutralizing agents. Models of guinea pigs and rabbits are more adequate, but the disadvantage is the high cost of the animals and their housing in the vivarium. 

In summary, we can say that the most common biomodels are either cheap and available, but not quite adequate, such as mice and to some extent rats, or are more adequate, but significantly more expensive, such as guinea pigs and rabbits. Such a situation led us to the desire to find another biomodel, which would be closer in cost to mice, but in terms of adequacy closer to that of guinea pigs.

We wondered if Syrian hamsters could be such a biomodel. Hamsters appear to be a promising laboratory animal for anthrax research. They are cheap, small, and do not require special conditions in the vivarium. For these reasons, hamsters can be used in large numbers in experiments. This is important for increasing the degree of the statistical reliability of the results obtained. At the same time, hamsters are larger than mice, and this increases the convenience of carrying out all manipulations with them. Nevertheless, despite these advantages, hamsters are rarely used for anthrax studies. Neither in the above-mentioned review [7] nor in the other literary sources we were able to find a sufficiently detailed description of the possibilities of anthrax research using a hamster biomodel.

Previously, some researchers have attempted to use hamsters to evaluate the efficacy of anthrax vaccines, but the results were not entirely consistent. Thus, Pomerantsev [8] reported on the possibility of effective vaccination of hamsters with a *B. anthracis* live vaccine strain. However, Fellows [9] vaccinated hamsters with the human anthrax vaccine AVA and found that although all vaccinated animals had high antibody titers, vaccination did not prevent death from infection with virulent *B. anthracis* strains. As a result, the question of whether the hamster model is suitable for studying anthrax vaccines remained controversial. We have tried to bring some clarity to this issue. In this work, we immunized three species of laboratory animals: C57BL/6 mice, outbred guinea pigs, and outbred Syrian (golden) hamsters with two recombinant proteins: rPA63 (protective antigen of *B. anthracis*) and the fusion protein rLF1PA4 containing the I domain of the protective antigen and the IV domain of the lethal factor. The effectiveness of vaccination was assessed by the challenge of the immunized animals with the encapsulated toxicogenic attenuated strain *B. anthracis* 71/12.

## 2. Materials and Methods

### 2.1. Bacterial Strains

To infect the vaccinated animals, we used strain *B. anthracis* 71/12, deposited in the SRCAMB collection. This strain is a subclone of the Tsenkowski II vaccine, and it has a plasmid profile of pXO1^+^ pXO2^+^.

The *E. coli* DH5α strain was used to construct the expression vectors; the *E. coli* BL21 strain was used to express the recombinant proteins.

### 2.2. Cloning

PA without native signal sequence (rPA63) was cloned into the commercial expression vector pET-SUMO (Champion™ pET SUMO Expression System, Thermo Fisher Scientific, Waltham, MA, USA). 

The fusion protein rLF1PA4, consisting of LF domain I and PA domain IV, was cloned into the commercial expression vector pLATE51 (aLICator LIC Cloning and Expression System, Thermo Fisher Scientific). The cloned fragments were generated by PCR using a template of *B. anthracis* 71/12 genomic DNA. All primers used for PCR are listed in Table 1. The PA63 locus was amplified using primers no. 10 and 8. The LF1PA4 locus was obtained by Gibson assembly of two fragments. One of them (LF 1 domain DNA) was obtained by three consecutive PCR reactions, in which the product of the first was used as a template for the second, and the product of the second was used as a template for the third. For these reactions, we used primers no. 1 and 4, 2 and 5, and 3 and 5, respectively. The second fragment (PA 4 domain DNA) was obtained in two sequential reactions using primers no. 6 and 8, and 7 and 9, respectively. The loci of the LF and PA genes obtained as a result of these PCR had regions complementary to each other and to the pLATE51 plasmid. This made it possible to use Gibson’s assembly of these fragments (with Gibson Assembly^®^ Cloning Kit, New England Biolabs, Ipswich, MA, USA) and to clone the product of this fusion into plasmid pLATE51 using LIC cloning (according to the manufacturer’s instructions).

Genomic DNA isolation was performed using the Genomic DNA Purification Kit (Thermo Fisher Scientific). Plasmid isolation was performed using a GeneJET Plasmid Miniprep Kit DNA (Thermo Fisher Scientific). Isolation of DNA fragments from agarose gel was carried out using the GeneJET Gel Extraction Kit (Thermo Fisher Scientific).

### 2.3. Biochemical Research Methods

The solubilization of recombinant proteins from inclusion bodies was carried out under denaturing conditions: the rLF1PAIV protein in the presence of 6 M urea and the rPA63 protein in the presence of 8 M urea.

Isolation and purification of proteins were carried out under denaturing conditions by chelating affinity chromatography using a Chelating Sepharose Fast Flow sorbent (GE Healthcare). The obtained protein fractions were analyzed by SDS-PAGE electrophoresis (10–12.5%) [10]. A set of standard proteins Spectra Multicolor Broad Range 260–10 kDa (Thermo Fisher Scientific) was used as molecular weight markers.

Fractions of the purified proteins were renatured by dialysis against 20 mM Tris (pH 7.9), and the denatured protein was removed by centrifugation at 10,000 rpm for 30 min. The protein concentration in the supernatant was assessed spectrophotometrically using an Ultrospec 3100 pro scanning spectrophotometer (Amersham), taking into account the molar extinction coefficients calculated for each protein.

For immunoblotting [11], Hybond P PVDF membranes (GE Healthcare, Chicago, IL, USA) and rabbit monospecific polyclonal antisera to native PA and LF at a dilution of 1: 100 were used. To detect bound rabbit antibodies, we used a conjugate of secondary goat antibodies to rabbit IgG (Sigma, Burlington, MA, USA) in a working dilution of 1:10,000 and a DAB (AppliChem, Council Bluffs, IA, USA).

Antibody levels in the sera of immunized animals were assessed by ELISA according to [12]. Protein antigens at a concentration of 5 μg/mL were adsorbed onto 96-well medium sorption plates (Greiner Bio-One, Frickenhausen, Germany), adsorption was performed overnight at 4 °C, then free binding sites were inactivated with a 1% BSA solution at 37 °C for 30 min.

Sera from immune and native animals were used in initial dilutions of 1:200, then titrated in 1:2 steps and incubated for 1 h at 37 °C. The serum of intact animals of the corresponding species was used as a control. Bound antibodies were detected with solutions of secondary goat antibodies conjugated with horseradish peroxidase in a working dilution of 1:10,000 and incubated for 1 h at 37 °C. Staining was carried out with a solution of orthophenylenediamine (OPD) with the addition of hydrogen peroxide to 0.03% for 20 min at room temperature in the dark. Then the reaction was stopped with a 0.1 M HCl. The optical density in the wells was measured at a wavelength of 492 nm on a Multiscan FC plate spectrophotometer (Thermo Fisher Scientific).

### 2.4. Animal Experiments

#### 2.4.1. Ethics Statement

All protocols for animal experiments were approved by the State Research Center for Applied Microbiology and Biotechnology Bioethics Committee (Permit No: VP-2021/1). They were performed in compliance with NIH Animal Welfare Insurance #A5476-01 issued on 02/07/2007 and the European Union guidelines and regulations on the handling, care, and protection of laboratory animals (https://eur-lex.europa.eu/eli/dir/2010/63/oj (accessed on 1 November 2021)).

A minimum number of animals was used for the experiments. The approved protocols provided scientifically validated humane endpoints, including pre-set criteria for the euthanasia of moribund mice via CO_2_ inhalation. In our study, the animals were euthanized when they became lethargic, dehydrated, moribund, unable to rise, or non-responsive to touch. The health conditions of the animals were monitored at least twice a day.

#### 2.4.2. Animals

Animals were purchased from the breading center “Stolbovaya” of the «Scientific Center of Biomedical Technology» of the Federal Medical-Biological Agency of Russia (https://www.pitomniki-stolbovaya.com (accessed on 1 November 2021)) and breading center “Andreevka” of the «Scientific Center of Biomedical Technology» of the Federal Medical-Biological Agency of Russia (http://andreevka.msk.ru/index.htm (accessed on 1 November 2021)). 

C57BL/6 mice (6–8 weeks old, 18–20 g), outbred Syrian (golden) hamsters (6–8 weeks-old, 80–100 g), and guinea pigs (6–8 weeks-old, 275 ± 25 g) of both genders were used in the experiments. Animals were housed in polycarbonate cages with space for comfortable movement (5 mice or 4 hamsters in a 484 cm^2^-cage and 3 guinea pigs in an 864 cm^2^-cage) with ad libitum access to food (Mouse Mixed Fodder PK-120, and Guinea Pig Mixed Fodder KK–122, Laboratorkorm, Russia) and tap water, under constant temperature and humidity conditions (22 ± 2 °C and 50 ± 10%, respectively) and a 12 h light/12 h dark cycle.

The general structure of the animal experiment is shown in Table S1.

#### 2.4.3. Immunization 

Mice (n = 100), guinea pigs (n = 80), and golden hamsters (n = 80) were immunized subcutaneously twice with an interval of 28 days. Animals were vaccinated subcutaneously in the inner part of the upper thigh. Purified recombinant proteins rLF1PA4 and rPA63 were injected with PBS at doses of 50 μg protein for mice and 100 μg protein for guinea pigs and hamsters. The dose volume was 100 μL for mice and hamsters and 250 μL for guinea pigs. Guinea pigs were vaccinated with twice the dose of recombinant proteins than mice due to their greater body weight and body size and the characteristics of their immune response. It was less clear which dose of recombinant anthrax proteins should be used to immunize hamsters. This information is not available at all since hamsters were almost never used in such studies. However, when immunizing hamsters with the human vaccine, AVA Fellows [9] used this vaccine at the same dosage that is commonly used for guinea pigs (this dose was higher than that used to immunize mice). Therefore, we also used the same dose of vaccine for hamsters as for guinea pigs. Proteins were administered both without additives and with complete (first immunization) and incomplete (second immunization) Freund’s adjuvant (FA) in a 1:1 volumetric ratio. One group of animals of each species was immunized only with complete (first immunization) and incomplete (second immunization) FA without recombinant proteins.

#### 2.4.4. Blood Sampling

At 14 days after the second immunization, blood samples were taken to determine the level of specific antibodies to the recombinant proteins rLF1PA4 and rPA63. In guinea pigs, blood was obtained from the marginal ear vein, in hamsters and mice, from the retroorbital sinus.

#### 2.4.5. Challenge

At 28 days after the second immunization, the animals were subcutaneously challenged with *B. anthracis* 71/12 strain. In total, five groups of immune animals of each species were challenged: two groups immunized with proteins rLF1PA4 and rPA63, two groups immunized with proteins with FA (rLF1PA4 + FA and rPA63 + FA), and one group that was administered only FA. Additionally, the sixth group of naive animals was used as a negative control.

Each group had four subgroups challenged with different doses. We used doses of 50 to 50,000 spores/animal for guinea pigs and Syrian hamsters (4 animals per subgroup), and 5 to 5000 spores/animal for C57BL/6 mice (5 animals per subgroup). Thus, the number of infected animals was: 96 guinea pigs (80 immune and 16 intact), 96 Syrian hamsters (80 immune and 16 intact), and 120 mice (100 immune and 20 intact).

We observed the animals for 21 days after infection; the surviving animals were humanely euthanized by CO_2_ inhalation. 

### 2.5. Statistics

The obtained data were statistically processed using the GraphPad Prism 7 program (https://www.graphpad.com/ (accessed on 1 November 2021)). Survival curves were plotted and analyzed using log-rank (Mantel–Cox) tests. In some cases, the levels of significance of differences (P) were determined using one-way ANOVA (Mann–Whitney). P values less than 0.05 were considered significant.

LD_50_ values were determined according to the method of Kärber, as modified by Ashmarin and Vorob’ov [13].

## 3. Results

### 3.1. Recombinant Protein-Producer Strains

It is known that the most immunogenic proteins of *B. anthracis* are the components of the anthrax toxin. First of all, this is PA. Antibodies to PA are necessary and sufficient for the formation of immunity to anthrax. The most immunologically active are PA domains I and IV [14]. LF also has specific immunogenicity. For example, it has been shown that the addition of biologically inactive LF or LF domain 1 to a vaccine containing PA can increase the protection [15]. Based on this, we decided to select the following proteins for immunization: (1) PA without a native signal sequence (PA63) and (2) a protein including the LF domain and PA IV, similar to the protein used in [16]. Since both proteins were not native but recombinant and expressed in a heterologous microorganism, we designated them as rPA63 and rLF1PA4, respectively. 

The genetic loci encoding these proteins were cloned into different plasmids: the rPA63 coding locus was cloned into the pET-SUMO plasmid, and the rLF1PA4 coding locus into the pLATE51 plasmid (Table 2). It was this combination of the cloned locus and the expression plasmid that allowed us to achieve the greatest yield of proteins. When *E. coli* strains carrying these plasmids were cultured in flasks under IPTG induction (2 mM), the yield of target proteins was more than 75 mg/L of culture. After cloning, we ordered from “Syntol” (Moscow) sequencing of plasmid regions that contain cloned genes to make sure that the expected gene sequences and their real sequences are identical. The result of the in silico translation of the nucleotide sequences is shown in Table 2.

**Figure 1:**
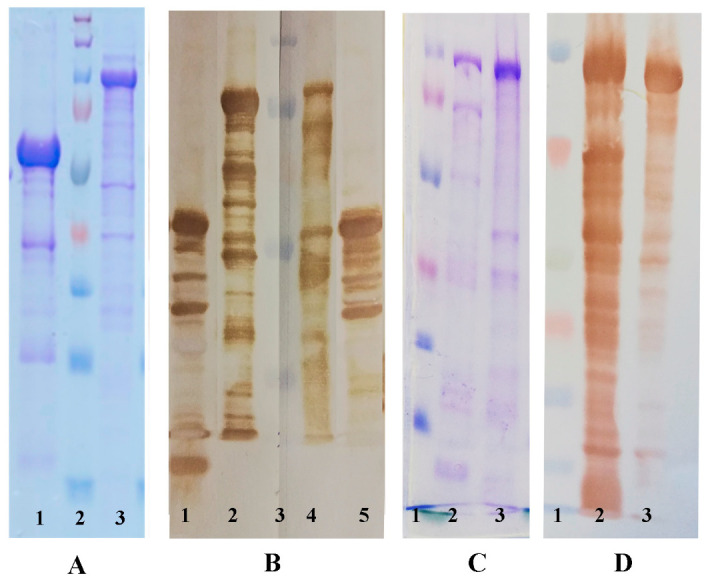
SDS-PAGE electropherogram and immunoblots of purified recombinant proteins. (The original figures are shown in Figure S1). (**A**). SDS-PAGE electropherogram: 1—renatured rLF1PA4; 2—Spectra Multicolor Broad Range Protein Ladder (260, 140, 100, 70, 50, 40, 35, 25, 15, and 10 kDa); 3—renatured rRA63; (**B**). immunoblot with rabbit antiserum to PA (left part, lane 1, 2) and with rabbit antiserum to LF (right part, 4,5): 1—renatured rLF1PA4; 2—native PA; 3—PageRuler Plus prestained Protein Ladder (250, 130, 95, 72, 55, 40, 36, 28, 17, and 10 kDa); 4—native LF; 5—renatured rLF1PA4. (**C**). SDS-PAGE electropherogram: 1—Spectra Multicolor Broad Range Protein Ladder (260, 140, 100, 70, 50, 40, 35, 25, 15, and 10 kDa); 2—native PA; 3—renaturated rRA63. (**D**). immunoblot with rabbit antiserum to PA. 1—Spectra Multicolor Broad Range Protein Ladder (260, 140, 100, 70, 50, 40, 35, 25, 15, and 10 kDa); 2—native PA; 3—renaturated rRA63.

The presence of a 6-His sequence at the N-terminus of the rLF1PA4 and rPA63 recombinant proteins allowed them to be purified using chelating affinity chromatography. As a result, protein preparations with a purity of 80–90% were obtained. Figure 1A show an electropherogram of rLF1PA4 and rPA63. In the rLF1PA4 preparation, we detected a single major band with an apparent molecular weight of 56.5 kDa (which is close to the calculated value of 53, 47 kDa) and a number of protein fragments, apparently formed during the hydrolysis of proteolytically unstable components of rLF1PA4 (Figure 1A). In the rPA63 preparation, we also detected one major band, with an apparent molecular weight of 98.7 kDa (which exceeds the calculated value of 80.75 kDa), and a number of minor protein fragments (Figure 1C). The immunological identity of the fragments of proteins rLF1PA4 and rPA63 and native LF and PA was confirmed by immunoblotting with rabbit monospecific polyclonal antisera to native PA and LF earlier obtained in accordance with the method described in [17]. We found that both proteins interact with rabbit anti-PA antiserum. rLF1PA4 also interacted with the serum to native LF, which confirms the presence of fragments of both LF and PA in rLF1PA4 fusion protein (Figure 1B,D).

We used the purified proteins rLF1PA4 and rPA63 to immunize mice, guinea pigs, and Syrian hamsters.

### 3.2. Study of Immunogenic Properties of rLF1PA4 and rPA63

**Figure 2:**
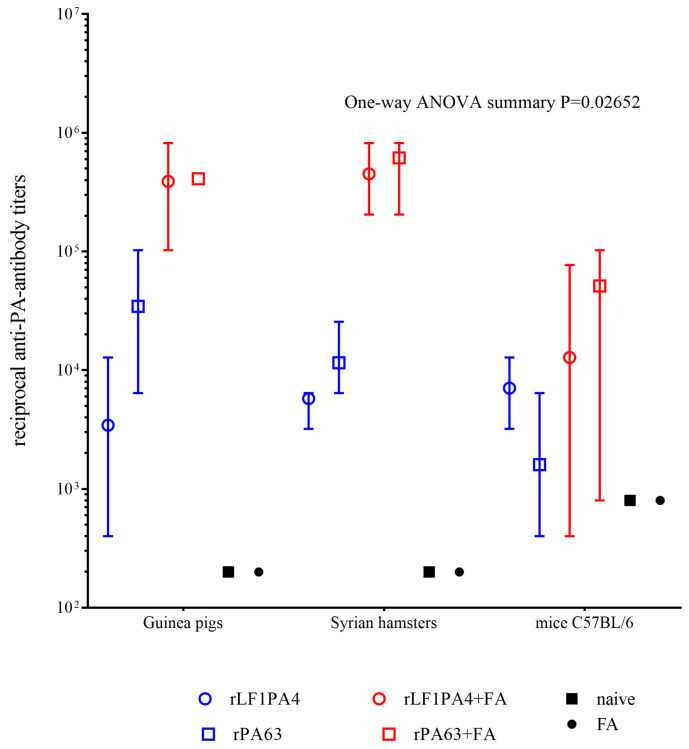
Reciprocal titers of antibodies to rPA63 and rLF1PA4 14 days after the second immunization. Graph was generated by GraphPad Prism 7 software. Mean values with upper and lower limits are indicated.

At 14 days after the second immunization, we took blood samples from animals in each group and measured the titers of antibodies to the rLF1PA4 and rPA63 in sera obtained from this blood by ELISA. The mean values of reciprocal antibody titers are presented in Table 3 and Figure 2. The significance of differences between groups was determined using one-way ANOVA.

As can be seen from the data presented (Figure 2), immunization of the animals led to the formation of a pool of specific antibodies in them. In vaccinated guinea pigs and Syrian hamsters, we found high antibody titers to the target proteins. The administration of proteins with added FA caused the expected statistically significant increase in titers by 1–2 orders of magnitude. Mice showed the greatest variation in individual antibody titer values, and the difference in antibody titer between groups, including the unvaccinated group, was insignificant in most cases.

### 3.3. Comparison of Immunization Protection for Different Animal Species

At 28 days after the second immunization, the animals were challenged with the *B. anthracis* 71/12 strain. The data on the death of animals and LD_50_ values are presented in Table 4.

**Figure 3:**
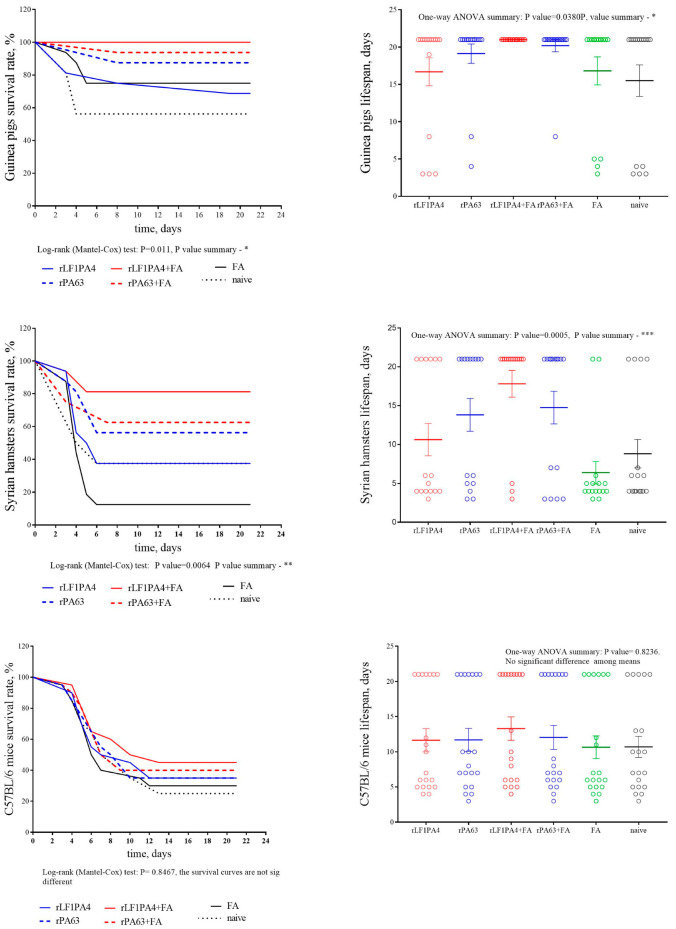
Survival curves and lifespan of guinea pigs, Syrian hamsters, and C57BL/6 mice immunized with recombinant proteins and intact. Graphs were generated by GraphPad Prism 7 software. Mean values with upper and lower limits are indicated. On the left, circles indicate each animal that died on a particular day of observation.

Based on the animal mortality data, we built survival curves and calculated the lifespan during the experiment for each group of animals (Figure 3). The lifespan during the experiment was calculated for each group considering the surviving animals, for which the lifespan was defined as 21 days (observation time). This indicator was used to more clearly demonstrate the differences in the number of surviving and dead animals between groups.

In our experiment, we observed that only groups of guinea pigs and hamsters were protected from challenge. Despite the fact that immunization in mice led to a slight increase in antibody titers, we did not find a protective effect of these antibodies. Upon challenge, all groups of immunized mice did not differ in terms of mortality among themselves, including the group that was injected with FA alone and the group of naive mice.

Guinea pigs were the most protected. In the group immunized with rLF1PA4 + FA, all infected animals survived. A high level of protection was observed in the groups immunized with rPA63, both with and without adjuvant. The group of guinea pigs immunized with rLF1PA4 without adjuvant was the least protected; mortality in this group did not differ from mortality in the FA group or the naive group. It should be noted that there was a relatively large survival rate (12 out of 16) among the guinea pigs injected with FA alone. Only animals that received the maximum infection dose (50,000 spores) died.

Syrian hamsters, although they were slightly less protected, showed the same trends as guinea pigs. Thus, a protective effect was recorded in Syrian hamsters in the three groups immunized with rLF1PA4 + FA, rPA63, and rPA63 + FA. The mortality rate of animals immunized with rLF1PA4 without FA did not differ significantly from the mortality rates in the FA group and in the unvaccinated control group. However, unlike guinea pigs, hamsters were not protected from infection by administering FA alone without immunogenic proteins. In the FA group, almost all hamsters died (14 out of 16); only one animal out of four infected with doses of 50 and 500 spores/animal survived. In the FA group, almost all hamsters died (14 out of 16); only two animals infected with minimal doses (50 and 500 spores) survived. Thus, we can say that the hamster model apparently allows minimizing the influence of the adjuvant on the final vaccine protection. This can be considered an advantage of the hamster model.

In general, we can see that the immunization of Syrian hamsters with the recombinant components of the anthrax toxin leads both to the production of specific antibodies (antibody titers are comparable to those in guinea pigs) and to protection from challenge with an encapsulated toxicogenic attenuated *B. anthracis* strain. In addition, the hamster model even showed some advantages over the guinea pig model. The influence of the adjuvant on the final result is less pronounced in hamsters than in guinea pigs (perhaps due to less stimulation of nonspecific immunity). Additionally, due to the relatively high virulence of the used strain for hamsters, they have a more pronounced difference in the survival rate of vaccinated and naive animals. 

Thus, a model of Syrian hamsters infected with an encapsulated toxicogenic attenuated strain of *B. anthracis* can be successfully used to assess the effectiveness of recombinant anthrax vaccines. This model, even if inferior to the guinea pig model, has significant advantages over the mouse model. Taking into account the low cost of hamsters, this model can be used instead of the mouse model as a first-line model in vaccine studies.

## 4. Discussion

As is widely known, it is the toxin that primarily affects the host organism during anthrax, and antibodies to the toxin (primarily to PA) are able to protect the host organism from lethal anthrax [16,18]. Therefore, the main task of vaccination is to lead to the formation of such antibodies, but at the same time not to cause serious damage to the macro-organism. Different approaches have been proposed for this at different times and in different countries. Historically, live attenuated strains were the first to be proposed as vaccines. They can be divided into two groups:

(1) Strains with a reduced copy number of the plasmid pXO1 and a low level of toxin expression, such as Pasteur II, Tsenkovsky II (variant spellings of the surname Tsenkovsky in the name of the vaccine in the English-language literature: Zenkowsky [19], Qiankefusiji [14] Russian: Цeнкoвcкий), and Rentian II [14];

(2) Single-plasmid strains lacking the pXO2 plasmid but retaining the ability to synthesize toxins, such as Sterne (a veterinary vaccine widely used all over the world) and STI-1 and A16R, which are human vaccines used in Russia and China, respectively [19].

Another approach seems to be safer: the use of the culture liquid of pXO1^+^ pXO2^−^ strains, adsorbed on aluminum compounds. These are vaccines such as AVA (anthrax vaccine adsorbed, USA) and AVP (anthrax vaccine precipitated, Great Britain). This technology also has its drawbacks; the main factor is composition instability, as the ratio of the components can vary from batch to batch. This problem could be solved, for example, by using recombinant anthrax toxin proteins for vaccination. This approach appears to be a rather promising idea, the implementation of which has been attempted for quite some time [15,16,19,20]. However, the main problem in the development of any anthrax vaccines is testing their efficacy. The fact is that, as we discussed in the introduction, the most common laboratory animal, mice, is not a completely adequate model for experimental anthrax. In nature, they are insensitive to alimentary infection with *B. anthracis* spores, but in the laboratory, infection with natural virulent strains is fatal for them even if they were vaccinated. Moreover, mice are highly susceptible to laboratory infection with pXO2^+^ strains, even if these strains are unable to synthesize the toxin. Despite this, mice are cheap, and some inbred lines are susceptible to infection with vaccine pXO1^+^ pXO2^−^ strains, which makes it possible to carry out the corresponding experiments not only in BSL3-4 laboratories; therefore, the mouse model remains quite common. Nevertheless, the mouse model’s peculiarities mean that the results obtained need to be confirmed in other animal species [7]. Thus, it may not be easy to prove, using only mice, that a vaccine under development is really worth the investment of resources and testing on more expensive animals. Therefore, the search for a cheap and affordable animal model that could replace or at least supplement the mouse model in anthrax vaccines research seems to be a rather urgent problem.

As we have shown, the Syrian hamsters would be such a “first-line” model in some cases. They are five times cheaper than guinea pigs and 15 times cheaper than rabbits. At the same time, their response to immunization with recombinant anthrax proteins is comparable to that of guinea pigs. Syrian hamsters, similar to guinea pigs, produce antibodies in comparable amounts, and these antibodies protect them from experimental infection.

It can be noted that we did not use a highly virulent natural strain for infection, but an attenuated two-plasmid strain 71/12, which is a subclone of the Tsenkovsky II vaccine. This strain has a slightly reduced virulence for laboratory animals (including hamsters). On the one hand, capsular toxicogenic vaccine strains such as Pasteur II and Tsenkovsky II differ little from natural strains in their biological properties; they act on the host organism with a full set of virulence factors and retain sufficient virulence for animals. On the other hand, the decrease in this virulence is sufficient for these strains to be considered a vaccine; therefore, working with them is possible with less biological safety measures than working with natural *B. anthracis* strains.

Therefore, strain 71/12 seemed to us convenient for assessing the effectiveness of anthrax vaccines on small animals; it does not require BSL3–4 conditions and, due to attenuation, does not lead to rapid and total death of animals, which allows us to register differences between groups.

Previously, Fellows et al. [9] reported that vaccination of hamsters with the AVA vaccine did not result in any protection. In light of our findings, we believe that our results and the results described in [9] do not contradict each other. Fellows used highly virulent natural strains that overcame the immunization of animals. We used an attenuated two-plasmid strain, which could not overcome the protection in immune hamsters and guinea pigs but led to the death of naive animals. Thus, it may be that recombinant anthrax vaccines cannot protect hamsters from infection with completely virulent natural strains of *B. anthracis*. This means that it is only possible to evaluate the effectiveness of recombinant vaccines using strains such as Pasteur II and Tsenkovsky II.

We can also put forward an alternative hypothesis to explain the discrepancy between our results and the results obtained by Fellows. It could be noted that both our study and Fellows used different vaccines to immunize hamsters. Fellows used the AVA vaccine, and we used the single-determinant protein vaccines. AVA is more complex and contains other toxin components (EF) and other secreted proteins. In theory, the presence of other proteins could reduce the AVA protectiveness against anthrax infection concretely for hamsters. This assumption is speculative and requires experimental testing using a wide variety of different anthrax vaccines in order to assess whether all of them are able to protect hamsters from infection. However, even if it turns out that hamsters in anthrax vaccine studies can only be used together with strains such as Tsenkovsky II, we believe that this model is still quite interesting.

To summarize, we can say that Syrian hamsters have thus far been an underestimated biological model for anthrax research. In this work, we have shown that hamsters can be used to assess the effectiveness of recombinant anthrax vaccines. A model of Syrian hamsters and their infection with two-plasmid vaccine strains may prove to be more useful than the traditional model of infecting immune mice with single-plasmid strains and could be much cheaper than guinea pig models.

## Figures and Tables

**Table 1:** Primers used in the work.

No.	Primer Name	Primer Sequence
1	G-LF1-F1	ATGGCGGGCGGTCATGGTG
2	G-LF1-F2Bam	ACAAGGGATCCATGGCGGGCGGTC
3	G-LF1-F3	GGTGATGATGATGACAAGGGATCCATGGC
4	G-LF1-R	CCGTTGATCTTTAAGTTCTTCCAAGGATAG
5	G-LF1-RAn	TGCGTTTAATTCCGCCCGTTGATCTTTAAGTTC
6	G-PA4-F1	GCGGAATTAAACGCAACTAACATATA
7	G-PA4F2An	GAACTTAAAGATCAACGGGCGGAATTAAACGC
8	G-PA4-R1Bam	GGATCCTTATCCTATCTCATAGCCTTTTTTAG
9	G-PA4-R2	GGAGATGGGAAGTCATTAGGATCCTTATCCTATCTCAT
10	PA63F1	TACTGGACCGATTCTCAAAATAAAAAAGAA

**Table 2:** Plasmids and the proteins encoded by them used in this work.

Plasmid	Protein Whose Gene Is Integrated into the Plasmid	In Silico Translated Amino Acid Sequence of a Recombinant Protein with a Leader Sequence Encoded by a Plasmid
pLATE51	LF1PA4	magshhhhhhgmasmtggqqmgrsgdddgsgagghgdvgmhvkekeknkdenkrkdeernktqeehlkeimkhivkievkgeeavkkeaaekllekvpsdvlemykaiggkiyivdgditkhislealsedkkkikdiygkdallhehyvyakegyepvlviqssedyventekalnvyyeigkilsrdilskinqpyqkfldvlntiknasdsdgqdllftnqlkehptdfsvefleqnsnevqevfakafayyiepqhrdvlqlyapeafnymdkfneqeinlsleelkdqraelnatniytvldkiklnakmnilirdkrfhydrnniavgadesvvkeahrevinssteglllnidkdirkilsgyiveiedteglkevindrydmlnisslrqdgktfidfkkyndklplyisnpnykvnvyavtkentiinpsengdtstngikkilifskkgyeig
pET-SUMO	PA63	mgsshhhhhhgsglvprgsasmsdsevnqeakpevkpevkpethinlkvsdgsseiffkikkttplrrlmeafakrqgkemdslrflydgiriqadqtpedldmedndiieahreqiggffvdstsagptvpdrdndgipdslevegytvdvknkrtflspwisnihekkgltkyksspekwstasdpysdfekvtgridknvspearhplvaaypivhvdmeniilsknedqstqntdsetrtiskntstsrthtsevhgnaevhasffdiggsvsagfsnsnsstvaidhslslagertwaetmglntadtarlnaniryvntgtapiynvlpttslvlgknqtlatikakenqlsqilapnnyypsknlapialnaqddfsstpitmnynqflelektkqlrldtdqvygniatynfengrvrvdtgsnwsevlpqiqettariifngkdlnlverriaavnpsdplettkpdmtlkealkiafgfnepngnlqyqgkditefdfnfdqqtsqniknqlaelnatniytvldkiklnakmnilirdkrfhydrnniavgadesvvkeahrevinssteglllnidkdirkilsgyiveiedteglkevindrydmlnisslrqdgktfidfkkyndklplyisnpnykvnvyavtkentiinpsengdtstngikkilifskkgyeig

**Table 3:** Values of reciprocal titers of antibodies to rLF1PA4 and rPA63 in the sera of experimental animals.

Antigens (Recombinant Proteins with or without Freund’s Adjuvant (FA))	Values of Reciprocal Titers (Min ÷ Max)		
	Guinea Pigs	Syrian Hamsters	C57BL/6 Mice
rLF1PA4	3440 (400 ÷ 12,800)	5760 (3200 ÷ 6400)	7040 (3200 ÷ 12,800)
rLF1PA4 + FA	389,120 (102,400 ÷ 819,200)	450,560 (204,800 ÷ 819,200)	12,800 (400 ÷ 76,800)
Increase in titers when adding FA to rLF1PA4 (times)	113	78	1.8
rPA63	34,560 (6400 ÷ 102,400)	11,520 (6400 ÷ 25,600)	1760 (800 ÷ 3200)
rPA63 + FA	409,600 (409,600 ÷ 409,600)	655,360 (204,800 ÷ 819,200)	51,200 (800 ÷ 102,400)
Increase in titers when adding FA to rPA63 (times)	11.8	56.8	29
FA without immunogenic proteins	<200	<200	<800
Unvaccinated control	<200	<200	<800

**Table 4:** Mortality rates and LD_50_ values after challenge of immunized and intact animals with *B. anthracis* 71/12.

Infection Dose	Antigens (Recombinant Proteins with or without Freund’s Adjuvant (FA))					
	rLF1PA4	rLF1PA4 + FA	rPA63	rPA63 + FA	FA	Naive
Guinea pigs (dead/animals in the subgroup)						
50	0/4	0/4	0/4	0/4	0/4	1/4
500	0/4	0/4	0/4	0/4	0/4	1/4
5000	2/4	0/4	0/4	0/4	0/4	1/4
50,000	3/4	0/4	2/4	1/4	4/4	4/4
LD_50_, CFU/animal, (min÷max)	8.8 × 10^3^ (228 × 10^3^ ÷ 7.1 × 10^4^)	−	>5 × 10^5^	>9 × 10^5^	1.6 ×10^4^ (5 × 10^3^ ÷ 1.6 × 10^5^)	2.8 × 10^3^ (7.1 × 10^2^ ÷ 1.4 × 10^5^)
Syrian hamsters (dead/animals in the subgroup)						
50	0/4	0/4	0/4	0/4	3/4	0/4
500	2/4	0/4	1/4	1/4	3/4	2/4
5000	4/4	0/4	3/4	1/4	4/4	4/4
50,000	4/4	3/4	3/4	4/4	4/4	4/4
LD_50_, CFU/animal, (min÷max)	5 × 10^2^ (1.3 × 10^2^÷2 × 10^3^)	2.8 × 10^5^ (1.4 × 10^5^ ÷ 7.1 × 10^6^)	2.8 × 10^4^ (7 × 10^2^÷1.4 × 10^5^)	5 × 10^3^ (1.3 × 10^3^ ÷ 3.2 ×10^4^)	50 (12 ÷ 199)	88 (22 ÷ 354)
C57BL/6 Mice (dead/animals in the subgroup)						
5	1/5	1/5	2/5	1/5	2/5	2/5
50	3/5	2/5	2/5	3/5	4/5	4/5
500	4/5	4/5	5/5	4/5	5/5	5/5
5000	5/5	5/5	5/5	5/5	5/5	5/5
LD_50_, CFU/animal, (min÷max)	39 (10 ÷ 158)	63 (16 ÷ 251)	25 (6 ÷ 100)	25 (6 ÷ 100)	10 (2.5 ÷ 40)	10 (2.5 ÷ 40)

## Data Availability

All data used for this study are available in the text of the article and in the Supplementary Materials.

## Supplementary Materials

The following are available online at https://www.mdpi.com/article/10.3390/life11121388/s1, Table S1: Animal experiment scheme; Figure S1: Original western blot figures of Figure 1.
