# Supplementary figures and images for: Using a Syrian (Golden) Hamster Biological Model for the Evaluation of Recombinant Anthrax Vaccines
Source: Life (Basel). 2021 Dec 11;11(12):1388. doi: 10.3390/life11121388 (PMC8704111; doi:10.3390/life11121388)

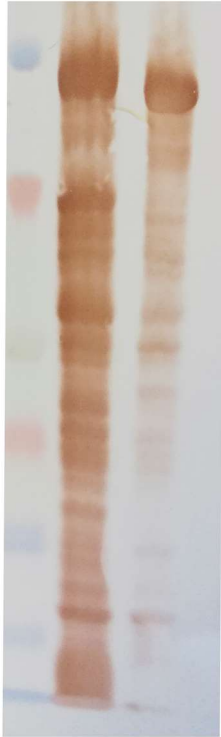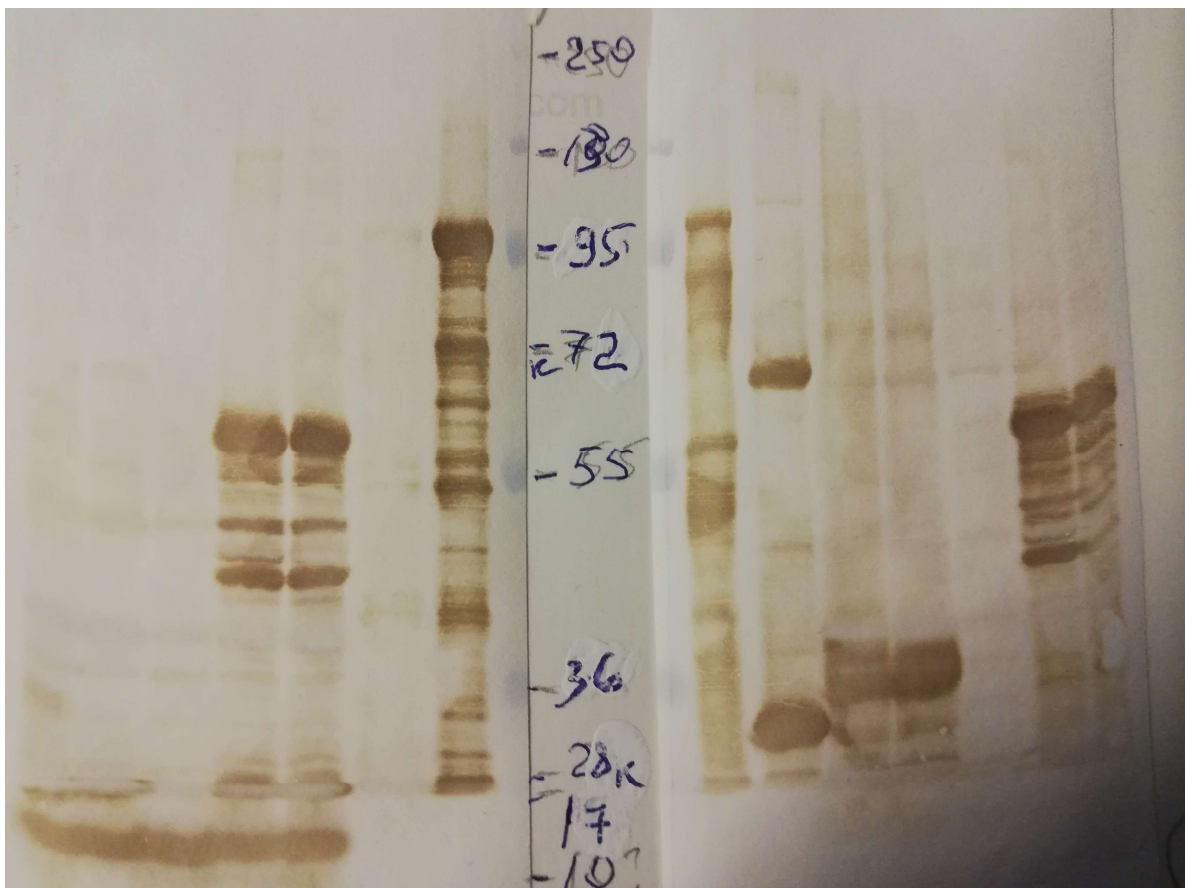

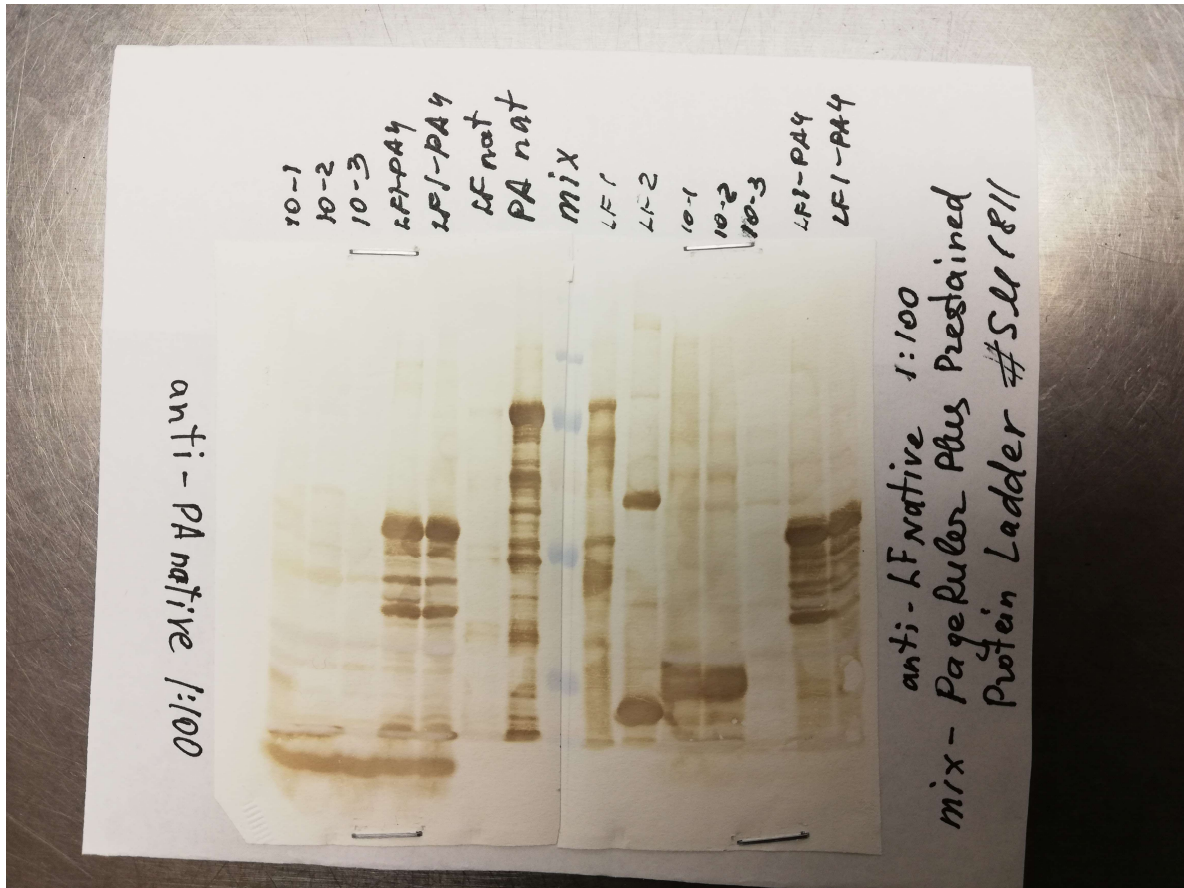

Figure S1. Original western blot figures of figure 1

Supplement: Supplementary file 1 [file life-11-01388-s001.zip › supplementary/life-1468992-supplementary-Figure S1.pdf]
